# Preparation and Immunochemical Characterization of a Water-Soluble Gluten Peptide Fraction for Improving the Diagnosis of Celiac Disease

**DOI:** 10.3390/nu16050742

**Published:** 2024-03-05

**Authors:** Niklas Meyer, Boris Illarionov, Markus Fischer, Herbert Wieser

**Affiliations:** Hamburg School of Food Science, Institute of Food Chemistry, University of Hamburg, 20146 Hamburg, Germanyboris.illarionov@uni-hamburg.de (B.I.); h.wieser2@gmx.de (H.W.)

**Keywords:** celiac disease, diagnosis, enzyme-linked immunosorbent assay, gluten, gluten immunogenic peptides, oral challenge

## Abstract

The diagnosis of celiac disease (CD) is complex and requires a multi-step procedure (symptoms, serology, duodenal biopsy, effect of a gluten-free diet, and optional genetic). The aim of the study was to contribute to the improvement of CD diagnosis by preparing a water-soluble gluten peptide fraction (called Solgluten) and by selecting gluten-specific enzyme-linked immunosorbent assays (ELISA) for the detection of gluten immunogenic gluten peptides (GIPs) in urine and blood serum spiked with Solgluten. Food-grade Solgluten was prepared by the extraction of a peptic digest of vital gluten with water, centrifugation, and freeze-drying. The process was relatively easy, repeatable, and cheap. The content of gliadin-derived GIPs was 491 mg/g. Solgluten was used as antigenic material to compare two competitive ELISA kits (R7021 and K3012) and two sandwich ELISA kits (M2114 and R7041) in their quality regarding the quantitation of GIPs in urine and blood serum. The quality parameters were the reactivity, sensitivity, coefficients of variation and determination, and curve shape. The evaluation of the kits showed a number of discrepancies in individual quality parameters measured in urine and serum. Due to the lowest limit of quantitation and the highest coefficient of determination, M2114 may be the first choice, while R7021 appeared to be less suitable because of the high coefficients of variation and unfavorable curve progression. The results set the stage for improving CD diagnosis by supplementing conventional blood tests with oral provocation with Solgluten and subsequent ELISA measurement of GIPs that could support the no-biopsy approach and by better assessing the effect of a gluten-free diet by monitoring adherence to the diet by measuring GIPs in urine and blood.

## 1. Introduction

Celiac disease (CD) is an inflammatory disease of the upper small intestine in genetically predisposed individuals that is triggered by gluten in the diet. According to the Codex Alimentarius, triggering gluten is defined as “a protein fraction from wheat, rye, barley, and oats or their crossbred varieties and derivatives that is insoluble in water and 0.5 mol/L NaCl” [1]. Gluten represents a complex protein mixture consisting of several hundred components. In the case of wheat, gluten proteins are divided into gliadins, soluble in aqueous alcohols (e.g., 60% ethanol), and insoluble glutenins. The gliadin fraction predominantly contains monomeric proteins classified as ω5-, ω1,2-, α-, and γ-gliadins, while the glutenin fraction consists of disulfide-linked protein aggregates formed by high-molecular-weight and low-molecular glutenin subunits (HMW- and LMW-GS) [2]. In vivo and in vitro studies have shown that all gluten protein types are involved in the formation of CD [3]. Structural features, unique to gluten proteins and causative for CD-specific immune reactions, are long repetitive amino acid sequences rich in glutamine and proline residues [2].

The overall features of CD have recently been reviewed by Caio et al. [4], Lindfors et al. [5], and Oxentenko and Rubio-Tapia [6]. Briefly, the global prevalence of CD has been estimated at around 1% of the general population. CD is genetically associated with the human leukocyte antigen (HLA) class II alleles HLA-DQ2, -DQ7, and -DQ8 at the major histocompatibility complex. Environmental factors (“second hits”) such as infections, imbalanced small intestinal microbiota, increased intestinal permeability, and infant feeding practices have been suggested to contribute to the onset of CD in addition to gluten and genetic predisposition. Symptoms associated with CD can be divided into gastrointestinal complaints such as chronic diarrhea, vomiting, and abdominal pain and extra-intestinal manifestations such as deficiencies of essential nutrients, dental manifestations, pregnancy complications, neurological disorders, and psychiatric complaints. Pathologically, CD is characterized by inflammation and damage of the small intestinal mucosa (“flattened mucosa”) and specific antibodies, targeted on gluten proteins (antigens) and tissue transglutaminase (autoantigen). Lifelong strict adherence to a gluten-free diet (GFD) is currently the only effective treatment of CD and its benefits for most patients are obvious.

CD is one of the most underdiagnosed disorders worldwide because diagnosis is complex and requires a multi-step procedure and a high level of clinical knowledge. A number of scientific societies, mainly from Europe and North America, have proposed appropriate guidelines for the diagnosis and management of CD [7]. In summary, the diagnostic scheme should consist of the following sequential approach: (1) clinical history and symptomatology; (2) serology; (3) small intestinal histology; (4) response to the GFD; and (5) genetic status. When patients, suspected to suffer from CD, have initiated a GFD before diagnosis has been set, gluten has to be reintroduced into the diet for a sufficient duration to reproduce serological abnormalities and characteristic intestinal damage [8].

Unfortunately, each step of the diagnostic scheme is fraught with specific problems. Regarding symptomatology, classical intestinal symptoms such as persistent diarrhea and abdominal pain usually prompt the physician to initiate a serological test but associations between extra-intestinal symptoms and CD are more difficult to detect [9]. In addition, a considerable portion of CD patients consists of asymptomatic individuals who may go unrecognized.

Serological testing is limited for the following reasons: the sensitivity and specificity of assays, usually used for diagnosis and targeted on endomysium, tissue transglutaminase, and deamidated gliadin peptides antibodies (EMAs, TGAs, and DGPAs), are quite variable in overall performance, making the accurate measurement of pathologic antibody levels difficult [10]. Moreover, the prevalence of seronegative CD accounts for up to 10% of CD cases, resulting in false-negative diagnoses [11]. In addition, most commonly applied assays are targeted at IgA class antibodies and are not helpful in the assessment of patients with IgA deficiency (around 7% of CD patients).

Major weaknesses of histological examination of the duodenum are the invasiveness of the method as well as the lack of uniformity in the reports of pathologists, the poor reproducibility and consistency in the classification of villous atrophy, and, last but not least, high costs [12]. Since 2012, guidelines of the European Society for Pediatric Gastroenterology, Hepatology, and Nutrition (ESPGHAN) have made it possible to avoid a biopsy in symptomatic pediatric patients with high levels of IgA TGAs (higher than 10 × the upper limit of normal (ULN)), positivity to IgA EMAs, and presence of HLA-DQ2/8 genes (“triple criteria”) [13]. Moreover, in a women who tests positive for TGA/EMA during pregnancy, the guidelines of the British Society of Gastroenterology suggest that the diagnostic strategy can avoid duodenal biopsy and that the procedure should be postponed to after the puerperium [14]. The “no biopsy approach” rule is still controversial and is not yet accepted in many parts of the world. The pros and cons of a biopsy-avoiding pathway and the contrast between pediatric and adult guidelines have been discussed by Raiteri et al. [7] and Reilly et al. [15].

Genetic testing based on HLA-DQ2/8 alleles cannot be used to confirm CD because they are quite common in the general population (around 30%). HLA-DQ testing is, therefore, used to rule out the existence of CD because they have high negative predictive values (around 97%) [16]. If a person suspected of having CD is negative for HLA-DQ2/8, CD is ruled out lifelong.

The clinical, serological, and histological responses to a strict GFD, finally used to confirm diagnosis, are highly variable and differ from patient to patient in a wide range. Slow responsiveness particularly occurs in those patients non-adherent to a strict GFD, which is why adherence to the GFD must be monitored. How to measure adherence to GFD is still controversial and requires further studies [17]. Recently, the determination of so-called gluten immunogenic peptides (GIPs) in feces and urine has been recommended by several groups to monitor the adherence of CD patients to the GFD (reviewed by Coto et al. [18]). GIPs can be defined as peptides that appear in blood, feces, and urine after ingestion of gluten-containing foods and can be quantified by gluten-specific enzyme-linked immunosorbent assays (ELISA). GIPs are derived from the repetitive amino acid sequences of gluten proteins during gastrointestinal digestion [2]. These sequence sections are rich in glutamine and proline, not degradable by gastrointestinal enzymes, and responsible for CD development. Up to now, most ELISA used for GIP detection have specificities only for GIPs derived from gliadins but digested glutenins also contain GIPs.

The first aim of the study presented here was to prepare and characterize a water-soluble gluten peptide fraction (called Solgluten in the following) obtained from the previously prepared peptic digest of vital gluten (Pepgluten) [19]. Solgluten is intended to be used for oral challenge tests as a supplementing tool for the diagnosis of CD. The second aim was to compare commercially available gluten-specific ELISAs in terms of their quality of detecting and quantifying GIPs in urine and serum samples spiked with different amounts of Solgluten. The combined results of the Solgluten preparation and characterization as well as ELISA evaluation should provide prerequisites for the improvement of step 2 (blood test), step 3 (no-biopsy approach), and step 4 (control of GFD compliance) within the diagnostic scheme of CD.

## 2. Materials and Methods

### 2.1. Reagents and Materials

All chemicals were purchased in analytical grade or higher. Wheat gluten (“vital gluten”), kindly provided by Hermann Kröner GmbH (Ibbenbueren, Germany), contained ≤8% moisture, ≤0.9% ash, and ≥78% protein (nitrogen × 6.25) according to the manufacturer’s product specification sheet. Pepsin capsules, offered as a dietary supplement (153 mg pepsin, corresponding to 3 × 10^6^ albumin digestion units, per capsule), were purchased from Dr. Clark Store (Chula Vista, CA, USA). The capsule coating (hypromellose) was removed and pure pepsin powder was used for gluten digestion. Sodium hydroxide (“baker’s brine”) was obtained from Minerva (Calbitz, Germany). Vinegar essence (Surig^®^, Speyer & Grund, Meerane, Germany; 25% acetic acid) and mineral water in a glass bottle were bought at a local supermarket. Acetic acid (pH 3.0) was prepared by mixing 900 mL of mineral water and 100 mL of vinegar essence. Water for analytical purposes was obtained with a Direct-Q 3 UV-R (Millipore, Schwalbach, Germany).

### 2.2. Urine and Blood Samples

Two healthy volunteers (one male aged 63 years and one female aged 27 years) were asked to maintain a GFD in the evening before providing urine samples the following morning. Informed consent was obtained from both participants. Around 200 mL of female urine and 400 mL of male urine were collected, divided into 10 mL portions, frozen, and stored at −20 °C. The human blood serum “off the clot” was provided by TCS biosciences LTD (Buckingham, UK). The donors were a 62 year old man and a 52 year old woman, both with the blood type B+. Serum was divided into 10 mL portions, frozen, and stored at −20 °C. On the morning of the ELISA measurements, urine and blood samples were thawed and spiked with Solgluten.

### 2.3. Preparation of Solgluten

To determine the repeatability of the preparation procedure, three assays (PREP 1, 2, and 3) were performed. Vital gluten (100 g) was digested with pepsin (4.5 g) in acetic acid (pH 3.0, 200 mL) at 37 °C for 120 min under magnetic stirring [19]. The digest was freeze-dried, dispersed in 200 mL of boiling mineral water, and heated for 10 min. After cooling to room temperature (RT ≈ 22 °C), the material was centrifuged (3000× *g*, 20 °C, 10 min) and the supernatant (extract 1) was decanted. The sediment was extracted stepwise twice with 100 mL of mineral water for 10 min at RT under magnetic stirring, centrifuged, and the supernatants were decanted (extracts 2 and 3). In the case of PREP 1, extracts 1, 2, and 3 were freeze-dried separately, weighed, and then combined. In the case of PREP 2 and PREP 3, extracts 1–3 were combined and then freeze-dried. The preparations were homogenized by using a coffee grinder (5 to 10 s pulses over 1 min) and incubated in a vacuum desiccator over baker’s brine until the odor of acetic acid had disappeared.

### 2.4. Basic Analysis

Moisture and ash contents of the preparations were determined according to the International Association for Cereal Science and Technology (ICC) Standard Methods 110/1 [20] and 104/1 [21], respectively. The nitrogen (N) content was analyzed using the Kjeldahl method. The N content was multiplied with a conversion factor of 5.76 to calculate the crude protein/peptide (CP) content. This unusual factor was used to obtain a more realistic value. The calculation of the factor was based on the proportions of gluten proteins (88.5% of *N*-containing components, factor 5.7) and pepsin plus albumins and globulins (11.5%, factor 6.25) previously determined in Pepgluten [19]. The protein/peptide patterns of vital gluten and Solgluten preparations were characterized by SDS-PAGE on a polyacrylamide-Bis-Tris gel (molecular mass of protein markers 14,400–116,000) according to the procedure presented by Wieser and Scherf [19]. The content of gliadin equivalents was determined according to ICC Standard 183 with a commercial competitive ELISA kit based on the R5 monoclonal antibody (RIDASCREEN^®^ Gliadin competitive, R-Biopharm, Darmstadt, Germany). Because the kit is intended to measure gliadin contents in the 20 mg/kg range and not in the >100,000 mg/kg range, the initial solution of Solgluten (2.7 mg/mL) was diluted by factor 20.

### 2.5. Quantitation of Water-Soluble Components of Pepgluten

Previously produced Pepgluten (100.0 mg) was weighed into a centrifuge tube and extracted stepwise three times with distilled water (1 mL) according to the analytical procedure developed for quantifying different protein fractions in wheat flour [22]. Each extraction step was performed by magnetic stirring for 20 min at RT. The suspensions were centrifuged for 20 min at RT and 6000× *g* and the supernatants were combined, freeze-dried, and weighed. The amount of extracted water-soluble components (Solgluten) was determined in triplicate.

### 2.6. Enzyme-Linked Immunosorbent Assay (ELISA)

All ELISA measurements were performed in a separate room and surfaces, vials, and equipment were cleaned with 60% ethanol to prevent gluten contamination. The following four commercially available ELISA test kits, developed for gluten detection in foods, were used (Table 1): RIDASCREEN^®^ Gliadin competitive; code R7021 (R-Biopharm, Darmstadt, Germany); GlutenTox ELISA competitive G12; code K3012 (Hygiena, Mississauga, ON, Canada); Wheat/Gluten (Gliadin) ELISA; code M2114 (Morinaga Institute of Biological Science, Yokohama, Japan); and RIDASCREEN^®^ Total Gluten; code R7041 (R-Biopharm, Darmstadt, Germany). Calibration was performed with the respective protein reference provided in each kit and calibration curves were constructed as stated in each kit. The concentrations of references used for calibration were the following: R7021: 10, 30, 90, and 270 ng/mL gliadin; K3012: 25, 125, 250, 500, and 1000 ng/mL gliadin; M2114: 0.78, 1.56, 3.13, 6.25, 13, 25, and 50 ng/mL wheat protein; and R7041: 5, 10, 20, 40, and 80 ng/mL gluten protein.

In preliminary experiments, the reactivity of the different kit antibodies towards the highest concentration of each kit reference (R7021, 270 ng/mL; K3012, 1000 ng/mL; M2114, 50 ng/mL; and R7041, 80 ng/mL) and the same concentrations of Solgluten dissolved in water were compared. The relative reactivity (absorbance units) was 40% (R7021), 65% (K3012), 32% (M2144), and 30% (R7041), respectively, related to the kit references (=100%). The resulting conversion factors 2.50 (R7021), 1.53 (K3012), 3.13 (M2114), and 3.33 (R7041) were used to generate Solgluten concentrations, the reactivity of which corresponded to those of the kit reference concentrations. In the main experiments, water, urine, and serum samples were spiked with Solgluten obtained from PREP 3 (content of gliadin equivalents: 492 mg/g). For this, the initial solution of Solgluten (27 mg/10 mL water) was stepwise diluted first with water and then with the sample dilution buffer provided in each kit in the last dilution step. Altogether, four Solgluten concentrations (expressed as measuring points MP 1–4), adapted to the reactivity of the kit references, were measured. The ELISA procedure was conducted in duplicates strictly according to the manufacturer’s instructions. The absorbance at 450 nm was read using the microplate reader SpectraMax M5e (Molecular Devices, LLC. San Jose, CA, USA). All measurements of references and Solgluten solutions were performed at one day per kit. The mean values of absorbance units (AU) were plotted versus the MP concentrations of kit references and Solgluten.

### 2.7. Data Analysis

Mean values, absolute standard deviations, and coefficients of variations (CV) were calculated for all quantitative data. The occurrence of outliers was examined by the Grubbs’s test. Microsoft Excel (Microsoft, Redmond, WA, USA) was used to assess significances of differences between the analytical parameters with one-way analysis (ANOVA) and Tukey’s test at *p* < 0.05. Values of the limit of detection (LOD) and the limit of quantitation (LOQ) were determined taking into account the DIN 32645 but without exclusion of outlier values from the calculations. The coefficient of determination (R^2^) was calculated using a 4 Parameter Logistic (4PL) nonlinear regression model. Origin Pro 2022 (OriginLab, Northampton, MA, USA) was used to plot the graphs.

## 3. Results

### 3.1. Preparation and Characterization of Solgluten

In a preliminary experiment, the amount of water-soluble components present in Pepgluten was determined by exhaustive extraction with distilled water according to the procedure previously developed for quantifying different protein fractions in wheat flour [22]. The yield of freeze-dried water-soluble components (Solgluten), obtained from 100 mg Pepgluten, was 80.3 ± 0.3 mg (n = 3), showing that starch (10.4% of Pepgluten), fat (4.7%), and dietary fiber (4.1%) [19] predominately remained in the insoluble residue. Thereafter, three preparative assays (PREP 1, 2, and 3) were conducted to study the performance and repeatability of the preparation procedure. The yields of Solgluten were 71.4% (PREP 1), 74.9% (PREP 2), and 70.3% (PREP 3) based on the amounts of gluten (100 g) and pepsin (4.5 g) used for the digestion (Table 2). The three single extraction steps applied for PREP 1 yielded 52.9% (E1), 12.6% (E2), and 5.9% (E3), showing that three steps were sufficient for exhaustive extraction. Considering the frequent transfers of solutions and substances during the process of digestion, heating, freeze-drying, and storage, the obtained yield (70–75%) appeared to be satisfactory, when compared to the analytical value (80%). The Solgluten powder obtained had a white-beige appearance, a slightly acidic smell, and a moderate bitter taste and yielded a clear solution when dissolved in water. Solgluten was stored in a closed vessel at −18 °C.

Compositional features of PREP 1, 2, and 3 were compared by quantitative analyses of CP, water, ash, and gliadin (GLIA) equivalents and by analytical SDS-PAGE. PREP 1 and 2 showed lower contents of CP (731 and 730 mg/g) compared to PREP 3 (755 mg/g) (Table 2). PREP 2 differed significantly from PREP 1 and PREP 3 in the content of GLIA equivalents (435 vs. 478 and 492 mg/g). Water content of PREP 1 (66 mg/g) was significantly higher than that of PREP 2 and 3 (51 and 52 mg/g). Obviously, PREP 1 was not dried for long enough. Ash contents of the preparations were similar (6.9–7.4 mg/g). In agreement with the previous characterization of Pepgluten, SDS-PAGE of PREP 1–PREP 3 concordantly demonstrated that gluten proteins were distinctively degraded by pepsin into peptides with relative molecular masses (M_r_) less than 14,400 (shown in Wieser and Scherf, 2018, Figure 1A [19]). The M_r_ ranged from 4900 to 14,000 (PREP 1), 9100 to 11,000 (PREP 2), and 7400 to 10,400 (PREP 3). Overall, the comparison of analytical data showed that the repeatability of the preparation process was satisfactory in terms of the yield and composition of Solgluten. PREP 3 was used for further experiments.

### 3.2. ELISA Measurements

Currently, more than 20 different ELISA kits for gluten analysis are on the market but each of these assays has its specific characteristics in terms of overall performance as well as specificity and sensitivity of the antibodies to different gluten protein epitopes [23]. For the present study, two competitive ELISAs (R7021 and K3012) and two sandwich ELISAs (M2114 and R7041) (Table 1) were selected to assess their suitability for quantifying GIPs in urine and serum. Competitive tests were the first choice because they enable the detection of both intact and degraded gluten proteins and the monoclonal antibodies R5 of R7021 and G12 of K3012 were already applied for quantitation of GLIA equivalents in urine and serum, respectively [18,24]. Sandwich ELISA M2114, including the polyclonal antibody pAb2, was repeatedly applied to quantify GIPs in serum [25,26,27] and, therefore, this test was also included into the study. The antibodies of the three selected kits are specific for gliadin- and not for glutenin-derived GIPs, although glutenins (HMW-GS and LMW-GS) also contain long repetitive sequences with immunogenic epitopes in both [28]. In order to capture glutenin peptides in addition to gliadin peptides, the recently developed sandwich ELISA R7041, containing monoclonal antibodies against LMW-and HMW-GS in addition to R5 against gliadins, was used as a fourth ELISA kit.

Primary aim of the immunochemical work was to evaluate the quality of the four kits regarding the quantitation of GIPs in water, urine, and serum samples spiked with defined amounts of Solgluten obtained from PREP 3. For calibration, protein references of the respective kits were used according to manufacturers’ protocols. In general, duplicate measurements at one day per kit were performed. Although inter-assay, inter-day, and inter-lab variabilities were not tested for full statistical evaluation, results should give valuable information on which assay is optimal for the detection of GIPs in urine and serum samples. A total of 101 measurements in duplicate were performed. The analytical targets included kit references as well as water samples, female (f) and male (m) urine, and serum samples spiked with Solgluten in four different concentrations each (MP 1–4). The resulting mean (AU and standard deviations are shown in Table 3. The results for key quality parameters of the kits related to GIP quantitation are summarized below. In particular, the performance of the assays, regarding urine and serum samples, was of interest in view of the future clinical applications of the kits.

**Table 3:** ELISA measurements (absorbance units (AU) ± standard deviation; n = 2) ^a^.

(A) Kit references							
**R7021**		**K3012**		**M2114**		**R7041**	
Conc. ^b^	AU	Conc. ^b^	AU	Conc. ^b^	AU	Conc. ^b^	AU
10	1.523 ± 0.093	25	0.536 ± 0.044	0.78	0.640 ± 0.037	5	0.328 ± 0.030
30	1.127 ± 0.023	125	0.441 ± 0.000	1.56	0.556 ± 0.311	10	0.625 ± 0.047
90	0.905 ± 0.086	250	0.336 ± 0.007	3.13	0.709 ± 0.091	20	0.902 ± 0.008
270	0.624 ± 0.024	500	0.278 ± 0.002	6.25	0.757 ± 0.056	40	1.890 ± 0.019
		1000	0.241 ± 0.036	13	1.010 ± 0.006	80	2.762 ± 0.001
				25	1.487 ± 0.025		
				50	2.028 ± 0.050		
(B) Solgluten solutions							
**Medium**	**Conc. ^c^**	**R7021**		**K3012**		**M2114**	**R7041**
Water	1	1.154 ± 0.064		0.554 ± 0.030		1.248 ± 0.835	1.643 ± 0.444
	2	0.792 ± 0.031		0.389 ± 0.035		1.457 ± 0.786	2.040 ± 0.361
	3	0.076 ± 0.004		0.315 ± 0.029		1.801 ± 0.020	2.425 ± 0.176
	4	0.071 ± 0.000		0.240 ± 0.013		2.330 ± 0.101	2.575 ± 0.054
Urine f	1	0.993 ± 0.170		0.617 ± 0.145		0.710 ± 0.377	1.568 ± 0.093
	2	0.602 ± 0.074		0.437 ± 0.012		0.766 ± 0.004	1.999 ± 0.095
	3	0.299 ± 0.127		0.377 ± 0.023		1.151 ± 0.321	2.303 ± 0.049
	4	0.259 ± 0.016		0.256 ± 0.047		2.293 ± 0.040	2.908 ± 0.016
Urine m	1	0.888 ± 0.158		0.635 ± 0.105		0.346 ± 0.018	1.394 ± 0.644
	2	0.717 ± 0.098		0.408 ± 0.025		0.745 ± 0.099	1.999 ± 0.140
	3	0.194 ± 0.021		0.311 ± 0.012		1.639 ± 0.017	2.196 ± 0.050
	4	0.058 ± 0.001		0.264 ± 0.022		2.152 ± 0.062	2.841 ± 0.029
Serum f	1	1.498 ± 0.360		0.551 ± 0.021		0.334 ± 0.028	0.635 ± 0.064
	2	1.460 ± 0.037		0.382 ± 0.021		0.566 ± 0.007	1.473 ± 0.035
	3	0.736 ± 0.170		0.296 ± 0.006		1.494 ± 0.034	2.163 ± 0.091
	4	0.109 ± 0.053		0.251 ± 0.016		2.142 ± 0.094	3.061 ± 0.037
Serum m	1	1.646 ± 0.310		0.584 ± 0.035		0.365 ± 0.013	0.983 ± 0.112
	2	1.097 ± 0.493		0.469 ± 0.101		1.124 ± 0.279	1.983 ± 0.401
	3	0.593 ± 0.105		0.269 ± 0.025		1.685 ± 0.092	2.288 ± 0.007
	4	0.197 ± 0.100		0.215 ± 0.004		2.209 ± 0.030	3.016 ± 0.068

^a^ f, female; m, male. ^b^ ng/mL. ^c^ Measuring points (see Figure 1).

#### 3.2.1. Reactivity

The reactivity of kit antibodies towards Solgluten peptides was determined by measuring the highest concentration of each kit reference (R7021, 270 ng/mL; K3012, 1000 ng/mL; M2114, 50 ng/mL; and R7041: 80 ng/mL) and the corresponding concentrations of Solgluten dissolved in water. The reactivity of the kit antibodies for Solgluten peptides, measured in AU, were 40% (R7021), 65% (K3012), 32% (M2114), and 30% (R7041) compared to kit references. Consequently, the antibodies of K3012 showed the highest binding capacity for Solgluten peptides, while those of M2114 and R2041 had the lowest affinity.

#### 3.2.2. Limit of Detection (LOD) and Quantitation (LOQ)

The results for LOD and LOQ of GIP measurements in urine and serum are presented in Table 4A. All in all, M2114 showed the lowest LOD (0.53–0.81 ng/mL) and LOQ (1.33–2.27 ng/mL) and R7021 showed the highest LOD (1.03–3.97 ng/mL) and LOQ (2.36–10.30 ng/mL). Most values of K3012 and R7041 were similar and in the middle range of both LOD (1.28–2.15 ng/mL) and LOQ (2.78–5.47 ng/mL). With one exception (LOQ of K3012, 1.03 ng/mL), differences in the mean LOD and LOQ between urine and serum samples were small (<0.24 and <0.19 ng/mL, respectively). The differences between female and male samples were enlarged in the mean LOD and LOQ of R7021 (2.94 and 7.94 ng/mL, respectively) and in the mean LOQ of K3012 (2.66 ng/mL), all other values were below 0.95 ng/mL. The differences between the kits, regarding LOD and LOQ (ng/mL), were well reflected by LOD and LOQ given the analysis of gluten-free foods (mg/kg) by the manufacturers (Table 1). LOD and LOQ are some of the most important criteria for GIP determination in the urine and serum of patients because of the low concentrations (ng/mL range) determined in these media [27,29]). Regarding this aspect, M2114 may be the first choice for applications in clinical investigations.

#### 3.2.3. Coefficient of Variation (CV)

The repeatability of measurements of spiked urine and serum samples, expressed as mean CV, is shown in Table 4B. The lowest mean CV (f + m) for urine samples was presented by R7041 (8.7%) followed by K3012 (10.7%), M2114 (13.2%), and R7021 (15.4%). With the exception of R7041, the CV of urine f samples was higher than that of urine m samples. Regarding serum samples, M2114 (6.5%), R7041 (6.6%), and K3012 (6.9%) provided highly acceptable mean CV, whereas R7021 showed a significantly higher non-acceptable value (28.9%). The CV of female serum samples was consistently lower compared to male serum samples. Considering all 16 measurements, R7041 (7.6%) provided the best and R7021 (22.1%) provided the worst repeatability.

#### 3.2.4. Coefficient of Determination (R^2^)

The results for the mean R^2^, determined for spiked urine and serum samples, are shown in Table 4C. Assuming that values >0.95 were considered acceptable, R7021 (urine m) and K3012 (serum f) each showed only one acceptable result. R7041 presented two (urine f and serum f) and M2114 presented three (urine m and serum f and serum m) acceptable values. With the exception of R7021, there was a trend that serum samples provided higher R^2^ values on average compared to urine samples. R^2^ values of serum f samples were generally higher than those of serum m samples, whereas R^2^ values of urine f samples were lower than those of urine m samples except R7041. In summary, M2114 showed the best total mean R^2^ (0.975) by far followed by R7041 (0.937), K3012 (0.923), and R7021 (0.922).

#### 3.2.5. Course of the Curves

**Figure 1:**
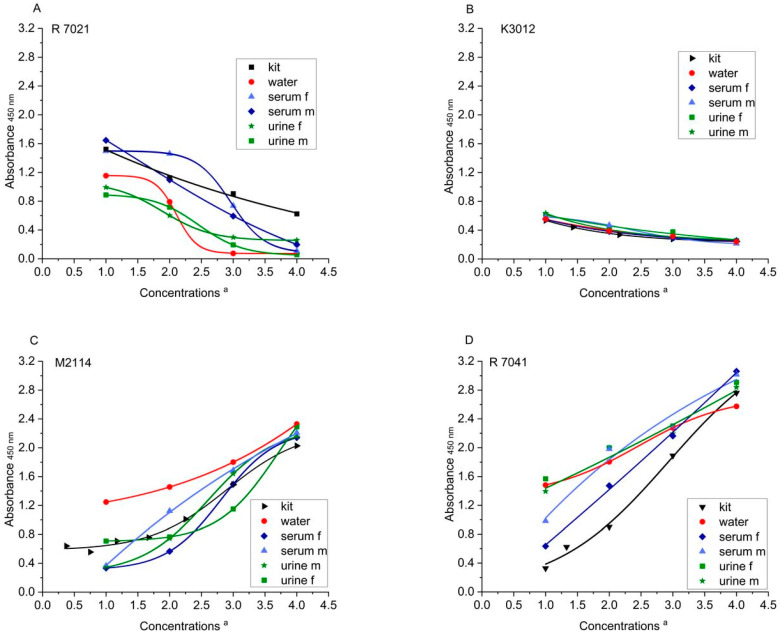
ELISA absorbances (λ = 450 nm) of kit references and Solgluten samples dissolved in water, urine, and serum ^a^. (**A**) R7021, (**B**) K3012, (**C**) M2114, and (**D**) R7041. ^a^ For concentrations of references see Table 3A. Concentrations of Solgluten (ng/mL): R7021: 25 (MP1), 75 (MP2), 225 (MP3), 675 (MP4); K3012: 38 (MP1), 306 (MP2), 765 (MP3), 1530 (MP4); M2114: 6.5 (MP1), 26.1 (MP2), 78.3 (MP3), 157.0 (MP4); R7041: 17 (MP1), 67 (MP2), 133 (MP3), and 266 (MP4).

In order to estimate the extent to which the curves of the spiked samples deviate from those of the respective kit reference (Figure 1A–D), the percentage AU of the samples were calculated in relation to the AU of the kit references (=100%) (four Solgluten concentrations each). Deviations below 75% and above 125% were considered as strongly deviating. Regarding R7021, all eight values of urine f and m samples, two values of serum m samples, and one value of serum f samples were <75%; one serum f sample exceeded 125% (Table 4D). The sample curves of K3012 mostly matched the reference curve, the exceptions were two urine f samples and one serum m sample (>125%). The curves resulting from M2114 deviated by <75% in one urine m sample, one serum m sample, and two serum f samples and by >125% in one urine f sample and one serum m sample. In addition, 7041 revealed two values >125% in urine f, serum f, and serum m samples each and three enhanced values of urine m sample. Including all 16 MP, R7021 had 12, R7041 had 9, M2114 had 7, and K3012 had 3 striking deviations from the corresponding kit references. Thus, the curves generated by K3012 largely matched those of the kit reference and the overall congruence of curves is impressive.

However, the reference curve of K3012 covered only a small range of AU (0.241–0.536; Table 3A) and, in consequence, the slope of the curve was unfavorably flat (Figure 1B). The other kits covered a considerably broader range of AU (R7021: 0.624–1.523; M2114: 0.640–2.028; R7041: 0.328–2.762) and the slope of the curves was more appropriate, particularly in the AU range >0.700. Altogether, the application of M2114 may be a compromise concerning course of curves.

Regarding the influence of media (water, urine, and serum) on the curve shape, M2114 showed the most significant deviations of both urine and serum samples from water samples characterized by consistently lower AU (Figure 1C). Curves of serum samples, generated by both R7021 (higher AU) (Figure 1A) and R7041 (lower AU) (Figure 1D) differed distinctly from that of water samples. Such clear effects could not be observed for K3012 (Figure 1B). When looking at the deviations of urine samples from serum samples, R7021 was characterized by a generally higher mean AU of serum samples, while K3012, M2114, and R7041 showed no fundamental differences. Evaluation of the gender effects on ELISA results for urine samples demonstrated that the four kits did not exhibit any noticeable differences. For serum samples, the mean AU of male samples measured with M2114 and R7041 was increased compared to female samples, while the mean AU of R7021 and K3012 did not differ significantly. Overall, R7021 showed the strongest influence of medium and gender among the ELISA kits tested.

#### 3.2.6. Resume

Regarding the quality of kits for urine analysis, M2114 (LOQ, R^2^, and course of curves) and K3012 (reactivity, CV, and course of curves) took up top positions. The low scoring criteria for reactivity and CV presented by M2114 and those for LOQ and R^2^ presented by K3012 appeared to be disadvantageous. R7041 was highly qualified in LOQ and CV but the other criteria were less acceptable. R7021 was characterized by insufficient quality in LOQ, CV, and the course of curves. The evaluation of the serum analyses showed best marks for M2114 in LOQ, CV, and R^2^ and for K3012 in reactivity and curve progression. R7041 displayed satisfactory quality in all tested criteria except reactivity, whereas R7021 showed non-acceptable results in LOQ, CV, and R^2^. All in all, M2114 may be the first choice for both urine and serum examination. If the high price of the M2114 kit (Table 1) will play a role for clinical application, K3012 may be an appropriate and cheaper alternative.

## 4. Discussion

### 4.1. Preparation and Properties of Solgluten

The first part of the work presented here involves the development of a procedure for the preparation of a water-soluble gluten peptide fraction (Solgluten) in food quality that is aimed to be used for diagnosis and clinical investigation of CD. Solgluten resembles the soluble peptic-tryptic gluten peptide fraction, called Frazer’s fraction FIII, presented in the late 1950s [30], which has been applied in innumerable in vivo and in vitro studies related to CD. Different from Frazer’s work, the combination of pepsin with trypsin was not used for digestion because gluten proteins include only a few amino acid residues (arginine and lysine) as cleavage sites for trypsin and cleavage is mostly blocked by neighboring proline [2] so that trypsin scarcely contributes to gluten digestion.

The quantitative data showed that exhaustive peptic digestion of gluten enables the almost complete transfer of highly insoluble gluten proteins to water-soluble peptides originating from all gluten protein types. Moreover, the results demonstrated that the procedure was characterized by satisfactory yields and sufficient repeatability. Due to cheap starting materials and relatively simple process steps, Solgluten can be produced at low cost. In comparison to Pepgluten, Solgluten is not only suitable for oral administration but also for experiments on tissues and cells in clinical and scientific studies because it completely dissolves in aqueous media. In future processes, the yield of peptides can be increased by using vital gluten with a higher content of crude protein (>80%). Because working steps (e.g., centrifugation and freeze-drying) are labor- and energy-intense, it should be considered as to whether one or two extraction steps are economically more worthwhile than three steps.

### 4.2. Quality of Gluten-Specific ELISAs

The second part of the work presented here aimed to compare four commercially available ELISA kits in their quality for GIP determination in urine and serum. Originally, gluten-specific ELISAs were developed for the control of gluten content in gluten-free foods but for several years, they have also been used for GIP detection in blood, feces, and urine to study intestinal permeability in wheat allergy and to monitor adherence to a GFD in CD. So far, however, ELISA systems selected for the detection of GIPs have been used without comparison and qualification of the assays available on the market, although ELISA results are known to be highly variable and depend, for example, on test formats, antibody specificities, the reference material used for calibration, and the food matrix [31].

Two different ELISA systems (sandwich and competitive) are currently used in food analysis [23]. The sandwich ELISA needs two binding sites of the antigen and is suitable for the detection of intact gluten proteins. The competitive ELISA needs only one binding site and is suitable for both intact proteins and peptides. The present work has shown that, in agreement with studies on GIPs in serum [25,26,27], sandwich ELISAs can be used for the quantitation of GIPs present in Solgluten as well as competitive ELISAs. The molecular masses of the peptides (up to 14,000) are large enough to be linked to the antibodies of the sandwich ELISAs used (M2114 and R7041). Regarding the quality of tests as a whole, the principal differences between competitive and sandwich assays could not be observed. Exceptions were the LOD and LOQ of sandwich assays that were lower in urine and serum samples compared to the competitive assays (Table 4A) and the distinctly higher reactivity of antibodies of the competitive tests towards Solgluten peptides. The use of antibodies against glutenins in addition to anti-gliadin antibodies (R7041) did not achieve any decisive advantages; the reactivity (30%) was even the lowest.

The comparative assessment of the kits showed a number of discrepancies in individual quality parameters. This result was not unexpected because of the different characteristics of the assays that cause systematic deviations. For example, when checking gluten-free food by ELISA, a number of problems were found in determining gluten in products with different matrices [32]. There was no single ELISA method that could accurately quantify gluten in all matrices. Similar to foods, urine and serum contain several hundred components, e.g., proteins, inorganic ions, and organic molecules that may hinder the efficient and reproducible binding of antibodies to GIPs. In the present study, the influence of media (water, urine, and serum) was most pronounced when R7021 was used. Indeed, the mean CV of the reference and water samples amounted to 5.4% and 3.5%, respectively, and corresponded to results obtained from food analysis. However, the mean CV determined for the urine samples was increased to 19.5% (f) and 11.2% (m) and, in the case of serum samples, even to 24.3% (f) and 33.1% (m) (Table 4B). Obviously, components of urine and serum act as interfering factors that do not allow satisfactory repeatability and thus statistical significance of the results. Further poor quality of R7021 was assessed for LOD and LOQ (Table 4A), R^2^ in serum (Table 4C), and the course of curves (Table 4D), which additionally suggests that R7021 is not qualified for providing accurate results concerning GIP contents in urine and serum.

The LOQ is surely the most important criterion for the reliable detection of GIPs in urine and serum, as extremely low concentrations were found here. For example, the urine of healthy adults on a normal gluten-containing diet contained 7–604 ng GIP/mL [29] and the challenge of healthy individuals with 2 g gluten resulted in urinary GIP concentrations between 1.7 and 9.1 ng/mL [33]. The LOQ of the ELISAs investigated (mean 1.8–6.2 ng/mL) may be sufficient to measure GIPs in urine but GIP concentrations found in serum are even lower than in urine. For example, levels in healthy adults exposed to 21 g of gluten proteins ranged from 0.015 to 0.659 ng/mL serum [24]. Data for CD patients with verified damage of duodenal mucosa, associated with an increased intestinal permeability, are not available and should be generated in future.

Due to the lowest LOQ (mean 1.8 ng/mL) and other high quality parameters, M2114 will be the first choice for urine and serum analysis. The usage of M2114 for serum analysis is supported by successful clinical studies in the field of wheat allergy [25,26,27]. The high kit price appears to be disadvantageous (Table 1) and, alternatively, K3012 can be used, however, with lower overall quality compared to M2114. Lateral flow immunoassay (LFIA) that uses the G12 antibody of K3012 (iVYCHECK GIP Urine, Biomedal S.L.) in combination with an LFIA reader [29] may be another tool to evaluate GIP concentrations in urine and possibly in serum and should be included in future investigations.

### 4.3. Improvement of CD Diagnosis

The use of Solgluten for oral challenge and the best ELISA for GIP detection may enable the improvement of CD diagnosis in several ways. First, blood tests after oral intake of Solgluten can be used to measure the GIP concentration as a marker of duodenal damage and increased intestinal permeability. Due to existing villous atrophy, it is to be expected that GIP concentrations in serum are significantly increased in patients with active CD. Thus, testing GIP levels together with testing CD-specific antibodies, e.g., against tissue transglutaminase, could strengthen the validity of serological examination (diagnostic step 2). Favorably, blood taken for antibody testing can also be utilized for GIP testing. The simple blood test, conducted after oral challenge with Solgluten, may be helpful in deciding on the no-biopsy approach. Together with the triple criteria proposed by ESPGHAN [13], possibly without the costly and labor-intensive EMA testing [34], significantly elevated GIP levels reflecting the damage of the small intestine would strengthen the argument for leaving out biopsies (diagnostic step 3). This could significantly reduce the burden on many patients as well as the number of endoscopy and the cost of CD diagnosis [35].

Second, persistent or recurring symptoms in spite of a GFD necessitate the control of dietary adherence (diagnostic step 4) and GIP measurement in feces and urine with gluten-specific ELISAs may be a valuable tool [18]. Regarding urine analysis, however, only one CD-specific antibody (G12), mostly applied in a lateral flow immunoassay, was used for monitoring dietary incompliance up to now. As a result, doubts have been expressed as to whether urinary GIP determination can be a reliable method for assessing compliance with the GFD [36]. Systematic investigations, including GIP analysis in urine and blood with the most suitable ELISA, should provide clearness on the usefulness of the procedure.

Thirdly, a number of patients, suspected to suffer from CD, have initiated a GFD before diagnosis has been set. Therefore, gluten-containing foods have to be reintroduced into the diet for a sufficient duration to reproduce serological abnormalities and characteristic intestinal damage [8]. This approach, however, implies the disadvantage that the physician cannot recognize in case of negative diagnostic findings, whether the patient has ingested sufficient amounts of gluten. In addition, patients are frequently unwilling to return to a gluten-containing diet. The controlled intake of defined amounts of Solgluten under perpetuation of a GFD, e.g., 5 g daily over a period of two weeks [37], appears to be a more convenient practice for patients and physicians.

### 4.4. Investigation of Intestinal Permeability

The condition of the small intestinal mucosa plays an important role in the pathogenesis of a number of diseases. For example, infections with intestinal pathogens, inflammatory bowel disease, irritable bowel syndrome, liver cirrhosis, and obesity as well as CD and food allergies have been associated with alterations in the intestinal barrier and increased intestinal permeability, respectively [38,39]. It is, therefore, important to determine the degree of intestinal permeability in patients with these disorders. In addition, external factors may provoke harmful intestinal alterations such as non-steroidal anti-inflammatory drugs, excessive alcohol consumption, high-intensity exercise, and psychological stress, for instance [40]. In these cases, it may also may be necessary to monitor intestinal permeability.

Intestinal permeability can be assessed through oral administration of non-digestible markers and their analysis in blood, specific organs, or urine. There are several types of markers excreted in an unaltered form: non-degradable sugars and sugar alcohols, radioisotopes, fluorescent-labelled dextrans, and polyethylene glycols [41]. The lactulose/mannitol (LAMA-) test that determines the ratio of lactulose to mannitol in urine after oral intake is the most common dual-sugar test used for the evaluation of small intestinal permeability. The main disadvantages of the test are the great variability in how the test is performed, the considerable heterogeneity in the results obtained, and the time-consuming and costly measurements. Moreover, results do not correlate with the permeability for macromolecules like bacterial toxins and food allergens [42] or gluten [37]. All in all, more robust alternative tests with a higher degree of clinical evidence are needed if measurements of intestinal permeability will find widespread clinical use.

It can be assumed that GIP concentrations in the urine and blood of patients suffering from conditions combined with a leaky bowel are significantly increased after gluten intake compared to healthy individuals. Oral challenge with Solgluten, followed by GIP analysis in urine or blood with the most suitable ELISA, may supplement or even replace other tests. Moreover, the innovative test may be used as non-invasive methods for the follow-up of healing processes during disease treatment and may replace endoscopy in many cases. After standardization, the new test can be used in all medical areas, where enhanced intestinal permeability occurs, for example, gastroenterology, allergology, pain medicine, drug medicine, and sport medicine.

## 5. Conclusions

In the first part of the study, the relatively simple and repeatable preparation of water-soluble gluten peptides (Solgluten) extracted from Pepgluten, the peptic digest of vital gluten [19], was demonstrated. Standard ELISA measurements revealed that the content of GLIA equivalents, the ingredient most important for oral challenge tests, was significantly enriched compared to Pepgluten (492 vs. 372 mg/g). Solgluten is not a novel material derived from gluten but is similar to Frazer‘s fraction FIII [30] that has been applied in numerous in vivo and in vitro studies on CD. Accordingly, Solgluten can be used for a variety of applications with regard to the diagnosis and investigations of CD. Due to the special preparation procedure that guarantees food-grade quality, Solgluten is particularly appropriate as a material for oral provocation tests.

The water-solubility of the preparation was essential for the examination of different commercially available ELISA kits concerning their quality to measure GIPs in urine and serum. As expected from previous applications in food analysis, single quality parameters of the four tested kits showed a number of discrepancies. Altogether, sandwich ELISA M2114 appears to be the most suitable tool to quantitate GIPs in both urine and serum. An example of the application is the control of the adherence to a GFD. The combination of oral challenge with Solgluten and analysis of GIPs in serum will support the conventional serological testing of CD patients to make the decision for a no biopsy approach easier. Moreover, oral challenge and GIP analysis in urine and/or serum may enable the investigation of an enhanced intestinal permeability occurring in a number of disorders and conditions.

## Figures and Tables

**Table 1:** Information on ELISA test kits provided by the manufacturers.

Name	RIDASCREEN^®^ Gliadin Competitive	GlutenTox ELISA Competitive G12	Wheat/Gluten ELISA Kit II	RIDASCREEN^®^ Total Gluten
Kit code	R7021	K3012	M2114	R7041
Company	R-Biopharm, Germany	Hygiena, Canada	Morinaga Inst., Japan	R-Biopharm, Germany
Format	competitive	competitive	sandwich	sandwich
Antibody ^a^	R5 mAb	G12 mAb	pAb2	four mAb ^b^
Main epitope	QQPFP ^c^	QPQLPY ^c^	several epitopes	several epitopes
LOD (mg/kg)	2.3 (gliadin)	1.6 (gliadin)	0.26 (gluten)	4 (gluten)
LOQ (mg/kg)	5 (gliadin)	2.5 (gliadin)	0.26 (gluten)	5 (gluten)
Cost ^d^	642	626	815	811

^a^ mAb, monoclonal antibody; pAb, polyclonal antibody. ^b^ R5, LMW1-, LMW2-, and HMW-glutenin antibodies. ^c^ One-letter-code for amino acids. ^d^ €, incl. tax.

**Table 2:** Composition ^a^ and yield of Solgluten.

Determinant	PREP 1	PREP 2	PREP 3
CP ^b^ [mg/g] (n = 5)	731.3 ± 3.6 ^A^	729.9 ± 2.4 ^A^	755.4 ± 3.4 ^B^
GLIA equivalents [mg/g] (n = 2)	478.0 ± 124.7 ^A^	435.4 ± 185.1 ^B^	491.7 ± 1.1^A^
Water [mg/g] (n = 5)	65.8 ± 0.3 ^A^	50.9 ± 0.4 ^B^	52.3 ± 0.4 ^B^
Ash [mg/g] (n = 5)	6.9 ± 0.1 ^A^	7.4 ± 0.2 ^A^	7.0 ± 0.4 ^A^
Yield (E1–E3) [%] ^c^	71.4 ^d^	74.9	70.3

^a^ Mean value ± standard deviation; different superscript letters (^A^ and ^B^) designate significant differences and the same letter (^A^ or ^B^) designates no significant difference between the samples (one-way analysis of variance (ANOVA), Tukey’s test, *p* < 0.05. ^b^ Crude protein/peptide (N × 5.76). ^c^ Based on vital gluten + pepsin. ^d^ E1–E3: 52.9 + 12.6 + 5.9.

**Table 4:** Quality parameters of ELISA Kits ^a^.

**(A) Limits of detection (LOD) and quantitation (LOQ) [ng/mL]**					
Medium		R7021	K3012	M2114	R7041
Urine f	LOD	2.22	1.33	0.53	1.54
Urine m	LOD	2.68	2.15	0.81	1.28
Mean	LOD	2.45	1.74	0.67	1.41
Serum f	LOD	3.97	1.37	0.55	1.35
Serum m	LOD	1.03	1.64	0.76	1.64
Mean	LOD	2.50	1.51	0.66	1.50
Urine f	LOQ	5.91	2.81	1.33	3.26
Urine m	LOQ	6.38	5.47	2.27	2.99
mean	LOQ	6.15	4.14	1.80	3.13
Serum f	LOQ	10.30	2.89	1.56	2.78
Serum m	LOQ	2.36	3.33	2.06	3.32
mean	LOQ	6.33	3.11	1.81	3.05
**(B) Coefficient of variation [%] (number of samples)**					
Medium		R7021	K3012	M2114	R7041
Urine f		19.5 (4)	12.7 (4)	20.8 (4)	3.3 (4)
Urine m		11.2 (4)	8.7 (4)	5.6 (4)	14.1 (4)
mean		15.4 (8)	10.7 (8)	13.2 (8)	8.7 (4)
Serum f		24.6 (4)	4.4 (4)	4.1 (4)	4.5 (4)
Serum m		33.1 (4)	9.4 (4)	8.8 (4)	8.6 (4)
mean		28.9 (8)	6.9 (8)	6.5 (8)	6.6 (8)
Total mean		22.1 (16)	8.8 (16)	9.8 (16)	7.6 (16)
**(C) Coefficient of determination (R^2^)**					
Medium		R7021	K3012	M2114	R7041
Urine f		0.931	0.839	0.929	0.987
Urine m		0.965	0.930	0.997	0.815
mean		0.948	0.885	0.963	0.901
Serum f		0.942	0.989	0.997	0.998
Serum m		0.868	0.933	0.975	0.949
mean		0.905	0.961	0.986	0.974
Total mean		0.922	0.923	0.975	0.937
**(D) Course of curves: number of AU deviations <75% and >125% related to kit reference (16 total MP each)**					
Medium		R7021	K3012	M2114	R7041
Urine f		4 (<)	2 (>)	1 (>)	2 (>)
Urine m		4 (<)	0	1 (<)	3 (>)
Serum f		1 (>), 1 (<)	0	2 (<)	2 (>)
Serum m		2 (<)	1 (>)	1 (>), 1 (<)	2 (>)
Σ		12	3,	6	9

^a^ f, female; m, male; MP, measuring point.

## Data Availability

The data presented in this study are available on request from the corresponding author. The data are not publicly available due to privacy reasons.

## References

[1] (2015). Codex Standard for Foods for Special Dietary Use for Persons Intolerant to Gluten. Revision 1.

[2] Wieser H., Koehler P., Scherf K.A. (2023). Chemistry of wheat gluten proteins: Qualitative composition. Cereal Chem..

[3] Wieser H., Koehler P., Konitzer K. (2014). Gluten—The precipitating factor. Celiac Disease and Gluten–Multidisciplinary Challenges and Opportunities.

[4] Caio G., Volta U., Sapone A., Leffler D.A., De Giorgio R., Catassi C., Fasano A. (2019). Celiac disease: A comprehensive current review. BMC Med..

[5] Lindfors K., Ciacci C., Kurppa K., Lundin K.E.A., Makharia G.K., Mearin M.L., Murray J.A., Verdu E.F., Kaukinen K. (2019). Coeliac disease. Nat. Rev. Dis. Prim..

[6] Oxentenko A.S., Rubio-Tapia A. (2019). Celiac Disease. Mayo Clin. Proc..

[7] Raiteri A., Granito A., Giamperoli T., Catenaro T., Negrini G., Tavoli F. (2022). Current guidelines for the management of celiac disease: A systematic review with comparative analysis. World J. Gastroenterol..

[8] Popp A., Laurikka P., Czika D., Kurppa K. (2023). The role of gluten challenge in the diagnosis of celiac disease: A review. Exp. Rev. Gastroenterol. Hepatol..

[9] Hujoel I.A., Reilly N.R., Rubio-Tapia A. (2019). Celiac disease: Clinical features and diagnosis. Gastroenterol. Clin. N. Am..

[10] Adriaanse M., Leffler D.A. (2015). Serum markers in the clinical management of celiac disease. Dig. Dis..

[11] Lebwohl B., Rubio-Tapia A., Assiri A., Newland C., Guandalini S. (2012). Diagnosis of celiac disease. Gastrointest. Endosc. Clin. N. Am..

[12] Villanacci V., Lorenzi L., Donato F., Auricchio R., Dziechciarz P., Gyimesi J., Koletzko S., Mišak Z., Laguna V.M., Polanco I. (2018). Histopathological evaluation of duodenal biopsy in the PreventCD project. An observational interobserver agreement study. APMIS.

[13] Husby S., Koletzko S., Korponay-Szabo P., Kurppa K., Mearin M.L., Ribes-Koninckx C., Shamir R., Troncone R., Auricchio R., Castillejo G. (2020). European Society Paediatric Gastroenterology, Hepatology and Nutritional Guidelines for diagnosing coeliac disease 2020. J. Pediatr. Gastroenterol. Nutr..

[14] Ludvigsson J.F., Bai J.C., Biagi F., Card T.R., Ciacci C., Ciclitira P.J., Green P.H., Hadjivassiliou M., Holdoway A., Van Heel D.A. (2014). Diagnosis and management of adult coeliac disease: Guidelines from the British Society of Gastroenterology. Gut.

[15] Reilly N., Husby S., Sanders D.S., Green P.H. (2018). Coeliac disease: To biopsy or not?. Nat. Rev. Gastroenterol. Hepatol..

[16] Lazar-Molnar E., Snyder M. (2018). The role of human leukocyte antigen in celiac disease diagnostics. Clin. Lab. Med..

[17] Wieser H., Ruiz-Carnicer A., Segura V., Comino I., Sousa C. (2021). Challenges of monitoring the gluten-free diet adherence in the management and follow-up of patients with celiac disease. Nutrients.

[18] Coto L., Mendia I., Sousa C., Bai J.C., Cebolla A. (2021). Determination of gluten immunogenic peptides for the management of the treatment adherence of celiac disease: A systematic review. J. Gastroenterol..

[19] Wieser H., Scherf K.A. (2018). Preparation of a defined gluten hydrolysate for diagnosis and clinical of wheat hypersensitivities. Nutrients.

[20] (1976). Determination of the Moisture Content of Cereals and Cereal Products (PRACTICAL Method).

[21] (1960). Determination of Ash in Cereals and Cereal Products.

[22] Wieser H., Antes S., Seilmeier W. (1998). Quantitative determination of gluten protein types in wheat by reversed-phase high-performance liquid chromatography. Cereal Chem..

[23] Scherf K.A., Poms R.E. (2016). Recent developments in analytical methods for tracing gluten. J. Cereal Sci..

[24] Scherf K.A., Lindenau A.C., Valentini L., Collado M.C., Garcia-Mantrana I., Christensen M., Tomsitz D., Kugler C., Biedermann T., Brockow K. (2019). Cofactors of wheat-dependent exercise-induced anaphylaxis do not increase highly individual gliadin absorption in healthy volunteers. Clin. Transl. Allergy.

[25] Matsuo H., Morimoto K., Akaki T., Kaneko S., Kusatake K., Kuroda T., Niihara H., Hide M., Morita E. (2005). Exercise and aspirine increase levels of circulating gliadin peptides in patients with wheat-dependent exercise- induced anaphylaxis. Clin. Exp. Allergy.

[26] Kohno K., Matsuo H., Takahashi H., Niihara H., Chinuki Y., Kaneko S., Honjoh T., Horikawa T., Mihara S., Morita E. (2013). Serum gliadin monitoring extracts patients with false negative results in challenge tests for the diagnosis of wheat-dependent exercise-induced anaphylaxis. Allergol. Int..

[27] Brockow K., Kneissl D., Valentini L., Zelger O., Grosber M., Kugler C., Werich M., Darsow U., Matsuo H., Morita E. (2015). Using a gluten oral food challenge protocol to improve diagnosis of wheat-dependent induced anaphylaxis. J. Allergy Clin. Immunol..

[28] Sollid L.M., Qiao S.W., Anderson R.P., Gianfrani C., Koning F. (2012). Nomenclature and listing of disease relevant gluten T-cell epitopes restricted by HLA-Q molecules. Immunogenetics.

[29] Moreno M.L., Cebolla A., Munoz-Suano A., Carrillo-Carrion C., Comino I., Pizarro A., León F., Rodríguez-Herrera A., Sousa C. (2017). Detection of gluten immunogenic peptides in the urine of patients with coeliac disease reveals transgression in the gluten-free diet and incomplete mucosal healing. Gut.

[30] Frazer A.C., Fletcher R.F., Ross C.A., Shaw B., Sammons H., Schneider R. (1959). Gluten-induced: The effect of partially digested gluten. Lancet.

[31] Diaz-Amigo C., Popping B. (2013). Accuracy of ELISA detection methods for gluten and reference: A realistic assessment. J. Agric. Food Chem..

[32] Bruins Slot I.D., Bremer M.G., van der Fels-Klerx I., Hamer R.J. (2015). Evaluation the performance of ELISA kits: The number do not tell the tail. Cereal Chem..

[33] Coto L., Sousa C., Cebolla A. (2021). Dynamics and considerations in the determination of the excretion gluten immunogenic peptides in urine: Individual variability at low gluten intake. Nutrients.

[34] Shiha M.G., Nandi N., Raju S.A., Wild G., Cross S.S., Singh P., Elli L., Makharia G.K., Sanders D.S. (2024). Accuracy of the no-biopsy approach for the diagnosis of coeliac disease in adults: A systematic review and meta-analysis. Gastroenterology.

[35] Shiha M.G., Nandi N., Hutchinson A.J., Raju S.A., Tai F.W., Elli L., Penny H.A., Sanders D.S. (2024). Cost-benefits and environmental impact of the no-biopsy approach for the diagnosis of coeliac disease in adults. Front. Gastroenterol..

[36] Monachesi C., Verma A.K., Catassi G.N., Franceschini E., Gatti S., Gesuita R., Lionetti E., Catassi C. (2021). Determination of urinary gluten immunogenic peptides to assess adherence to the gluten-free diet: A randomized, double-blind, controlled study. Clin. Transl. Gastroenterol..

[37] Leffler D., Schuppan D., Pallav K., Najarian R., Goldsmith J.D., Hansen J., Kabbani T., Dennis M., Kelly C.P. (2013). Kinetics of the serological and symptomatic responses to gluten challenge in adults with coeliac. Gut.

[38] König J., Wells J., Cani P.D., Garcia-Rodenas C.L., MacDonald T., Mercenier A., Whyte J., Troost F., Brummer R.J. (2016). Human intestinal barrier function in health and disease. Clin. Trans. Gastroenterol..

[39] Camilleri M. (2019). Leaky gut: Mechanisms, measurement and clinical implications in humans. Gut.

[40] Vancamelbeke M., Vermeire S. (2017). The intestinal barrier: A fundamental role in health and disease. Rev. Gastroenterol. Hepatol..

[41] Vanuytsel T., Tack J., Farre R. (2021). The role of intestinal permeability in gastrointestinal disorders and current methods of evaluation. Front. Nutr..

[42] Voidani A. (2013). For the assessment of intestinal permeability, size matters. Altern. Ther..

